# Autotrophic biofilms sustained by deeply sourced groundwater host diverse bacteria implicated in sulfur and hydrogen metabolism

**DOI:** 10.1186/s40168-023-01704-w

**Published:** 2024-01-26

**Authors:** Luis E. Valentin-Alvarado, Sirine C. Fakra, Alexander J. Probst, Jonathan R. Giska, Alexander L. Jaffe, Luke M. Oltrogge, Jacob West-Roberts, Joel Rowland, Michael Manga, David F. Savage, Chris Greening, Brett J. Baker, Jillian F. Banfield

**Affiliations:** 1 Graduate Group in Microbiology, University of California, Berkeley, CA USA; 2 Innovative Genomics Institute, University of California, Berkeley, CA USA; 3 Advanced Light Source, Lawrence Berkeley National Laboratory, Berkeley, CA USA; 4 Earth and Planetary Science, University of California, Berkeley, CA USA; 5 Environmental Metagenomics, Research Center One Health Ruhr of the University Alliance Ruhr, Faculty of Chemistry,, University of Duisburg-Essen, Essen, Essen, Germany; 6 Cleaner Air Oregon Program, Oregon Department of Environmental Quality, Portland, USA; 7 Department of Molecular and Cell Biology, University of California, Berkeley, CA USA; 8 Howard Hughes Medical Institute, University of California, Berkeley, CA 94720 USA; 9 Environmental Science, Policy and Management, University of California, Berkeley, CA USA; 10 Earth and Env. Sciences Division, Los Alamos National Laboratory, Los Alamos, NM USA; 11 University of Duisburg-Essen, Universitätsstraße 5, 45141 Essen, Germany; 12 Department of Microbiology, Biomedicine Discovery Institute, Monash University, Clayton, Australia; 13 Department of Integrative Biology, University of Texas, Austin, USA; 14 Department of Marine Science, University of Texas, Austin, USA; 15 Energy Geoscience Division, Lawrence Berkeley National Laboratory, Berkeley, CA USA

**Keywords:** Candidate phyla radiation, Groundwater microbiome, Synchrotron-based spectromicroscopy

## Abstract

**Background:**

Biofilms in sulfide-rich springs present intricate microbial communities that play pivotal roles in biogeochemical cycling. We studied chemoautotrophically based biofilms that host diverse CPR bacteria and grow in sulfide-rich springs to investigate microbial controls on biogeochemical cycling.

**Results:**

Sulfide springs biofilms were investigated using bulk geochemical analysis, genome-resolved metagenomics, and scanning transmission X-ray microscopy (STXM) at room temperature and 87 K. Chemolithotrophic sulfur-oxidizing bacteria, including *Thiothrix* and *Beggiatoa*, dominate the biofilms, which also contain CPR Gracilibacteria, Absconditabacteria, Saccharibacteria, Peregrinibacteria, Berkelbacteria, Microgenomates, and Parcubacteria. STXM imaging revealed ultra-small cells near the surfaces of filamentous bacteria that may be CPR bacterial episymbionts. STXM and NEXAFS spectroscopy at carbon K and sulfur L_2,3_ edges show that filamentous bacteria contain protein-encapsulated spherical elemental sulfur granules, indicating that they are sulfur oxidizers, likely *Thiothrix*. Berkelbacteria and Moranbacteria in the same biofilm sample are predicted to have a novel electron bifurcating group 3b [NiFe]-hydrogenase, putatively a sulfhydrogenase, potentially linked to sulfur metabolism via redox cofactors. This complex could potentially contribute to symbioses, for example, with sulfur-oxidizing bacteria such as *Thiothrix* that is based on cryptic sulfur cycling. One Doudnabacteria genome encodes adjacent sulfur dioxygenase and rhodanese genes that may convert thiosulfate to sulfite. We find similar conserved genomic architecture associated with CPR bacteria from other sulfur-rich subsurface ecosystems.

**Conclusions:**

Our combined metagenomic, geochemical, spectromicroscopic, and structural bioinformatics analyses of biofilms growing in sulfide-rich springs revealed consortia that contain CPR bacteria and sulfur-oxidizing Proteobacteria, including *Thiothrix*, and bacteria from a new family within Beggiatoales. We infer roles for CPR bacteria in sulfur and hydrogen cycling.

Video Abstract

**Supplementary Information:**

The online version contains supplementary material available at 10.1186/s40168-023-01704-w.

## Background

Sulfur is the fifth most abundant element on earth, and the sulfur cycle is a key component of Earth’s interlinked biogeochemical cycles [1, 2]. In natural ecosystems, sulfur exists in several oxidation states, − 2, 0, + 2, + 4, and + 6 being the most common, in the forms of polysulfide (HS_x_ or S_x_^2−^; − 2, 0), thiosulfate (S_2_O_3_^2−^; − 1, + 5), tetrathionate (S_4_O_6_^2−^; − 2, + 6), sulfite (SO_3_^2−^; + 4), and sulfate (SO_4_^2−^; + 6). Microbes play an important role in sulfur cycling in aqueous and soil environments. H_2_S is a toxic compound that must be maintained at low levels for the sustained growth of microbial consortia; thus, microbial sulfide oxidation is beneficial at the community level.

Sulfide (S^2−^) is common in natural springs and can serve as a source of energy and reducing power for chemolithoautotrophic microorganisms. Chemolithoautotrophic microbial communities with members that carry out the oxidation, reduction, and disproportionation of sulfur compounds are found in environments such as hydrothermal vents [3, 4], water column oxic/anoxic interfaces [5–7], terrestrial caves [8–10], groundwater [11, 12], and activated sludges [13]. Beggiatoaceae and Thiotrichaceae that have been cultivated have been shown to use hydrogen sulfide either mixotrophically or heterotrophically [14–17]. *Beggiatoa* spp. are gliding filamentous bacteria that form intracellular spherical S^0^ granules that may oxidize to sulfate when H_2_S supply becomes limited [18]. *Thiothrix* spp. are gliding bacteria that can grow as long multicellular filaments (cells in a microtubular sheath), can form rosettes, and are known to accumulate intracellular spherical S^0^ granules when in the presence of reduced sulfur compounds [13, 19] and organics or CO_2_ (carbon and energy source) [14]. Prior work [20–24] indicates that sulfur-oxidizing bacteria support communities by providing resources such as fixed carbon and nitrogen.

To date, most studies of sulfur-based chemoautotrophic ecosystems have investigated the roles of the relatively most abundant organisms. However, it is well understood that microbial biofilms are structured as networks of interacting organisms, some of which are fundamentally dependent on other community members. Of particular interest are candidate phyla radiation (CPR) bacteria (also known as Patescibacteria) [25–28] that can form symbioses with host organisms [29–31]. Prior surveys have documented CPR bacteria in sulfur-based communities [25, 32, 33], yet the nature of CPR-host relationships and the roles of CPR in sulfur-based communities remain under-explored.

Here, we studied chemoautotrophic microbial communities sustained by sulfur metabolism in two mineral springs MS4 and MS11 [34] at Alum Rock Park, CA, USA, where sulfide-rich groundwater discharges along the Hayward fault. We profiled oxygen isotopes, temperature, water composition, and spring discharge rates to constrain the sources of water. We then further combined genome-resolved metagenomics with X-ray spectromicroscopy and scanning electron microscopy to investigate metabolic capacities, interdependencies, and structure of the microbial biofilm community at these two springs. Synchrotron-based spectromicroscopy revealed a close association between ultra-small cells and sulfur-oxidizing bacteria. The possibility that these bacteria are CPR might indicate the existence of a cryptic chemoautotrophic ecosystem. We predict the contributions of the major community members to carbon, nitrogen, sulfur, and hydrogen cycling and investigate the potential roles of the abundant and diverse CPR bacteria in these consortia.

## Materials and methods

### Site description and microbial biomass collection

**Fig. 1:**
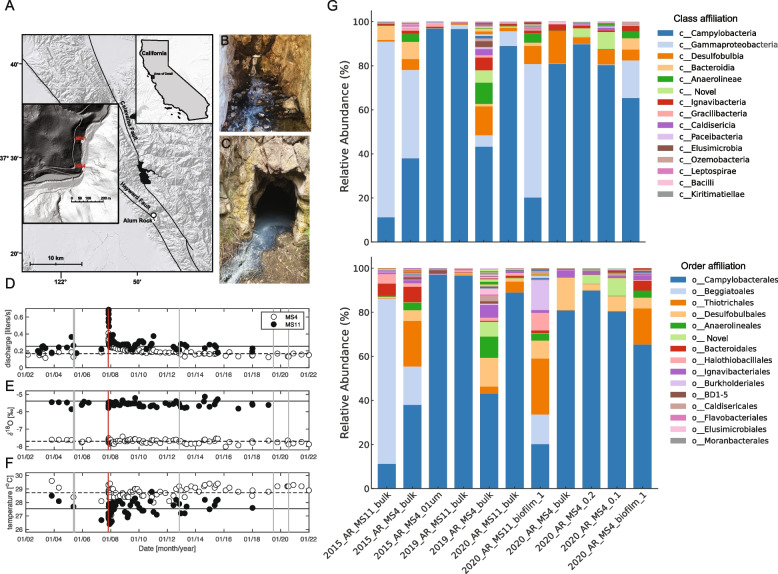
**A** Shaded relief map showing the location of Alum Rock springs, CA, USA. Insets show the location of Alum Rock and of the MS4 and MS11 springs. Photographs of (**B**) MS4 and (**C**) MS11 biofilms. Thin white streamers (5–10 cm) are mostly found attached to the surfaces of rocks. Hydrogeological properties (**D**) discharge, (**E**) δ^18^Ο, and (**F**) temperature are steady over periods greater than a decade, except following large regional earthquakes. A discharge increase in late 2007 followed a magnitude 5.6 earthquake with an epicenter 4 km from the springs (vertical red line), neither δ^18^Ο nor the temperature changed indicating that fluid sources did not change. The horizontal lines show averages of plotted quantities over the entire sampling period, except discharge for which the average excludes the first 2 years after the earthquake. Vertical gray lines show dates of biofilm and planktonic sampling. **G** Microbial community composition at the class and order levels, respectively, highlighting the top 15 most abundant groups in each category. Each bar represents a sample collected from different biotopes (bulk/biofilm, 0.1-µm filter) in the MS4 and MS11 springs over several years (2015, 2019, and 2020). The stacked bar plots illustrate the relative abundance of each microbial group, with each color corresponding to a different group, from top to bottom in decreasing order of overall abundance across all samples

The spring system is located along Penitencia Creek in Alum Rock Park, San Jose, CA, USA (37°23′57.7″N, 121°47′48.8″W) (Fig. 1A). The two sample sites, Mineral Springs 4 and 11 (MS4 and MS11), are located on opposite sides of the creek approximately 250 m from one another (Fig. 1B, C). Samples for geochemical analyses were taken in May 2005, during the dry season, and were filtered on-site using sterile 0.2-µm filters. Biofilm samples for scanning electron microscopy were collected from both sites using sterile pipettes. Samples were then transported back to the laboratory on ice, and solutions for cation analyses were acidified with 3% nitric acid. Biofilm samples for metagenomic sequencing were collected on June 13, 2015, July 2, 2019, and July 24, 2020. Planktonic samples were collected on June 10, 2015 and July 24, 2020. Two sets of planktonic samples were taken by sequentially filtering 379 L and 208 L of water, respectively, from the MS4 spring onto 0.65-μm and 0.1-µm large volume filters (GraverTech 5-inch ZTEC-G filter). Filters were frozen on dry ice at the site and stored at − 80 °C for genome-resolved metagenomic analyses. For synchrotron-based measurements (STXM and X-ray microprobe), thin white streamers were collected on June 13, 2015, with sterile tweezers at both sites and flash-frozen on site. A 2-µL droplet of biofilm sample was deposited using a sterile pipette onto either a 3-mm diameter Si_3_N_4_ window (SiMPore) or a TEM Cu grid (300 mesh, lacey carbon coated formvar, Ted Pella Inc.).  The grids were manually blotted with filter paper (Grade 1 filter paper, Whatman®) and immediately plunged into liquid nitrogen using a portable LN_2_ plunger, and gas ethane (used for cryo-plunging) was not available at the time of sampling. Samples were not rinsed or spinned so as to preserve the structural integrity of the filaments and preserve the CPR bacteria-bacteria-filaments spatial relationships. Samples were stored in a LN_2_ storage dewar (Taylor-Wharton, 34 L) until measurements.

### Geochemical analysis

Water discharge (volume/time) was measured by diverting water into either a bucket or graduated cylinder to measure volume, and time was recorded with a stopwatch. Temperature was measured with a type K thermocouple until February 2008 and thereafter with a thermistor. Accuracy is 0.2 °C and 0.1 °C, respectively. Water for O and H isotope measurements was collected in 250-mL Nalgene bottles. Discharge and temperature were not measured if outflow channels from the springs backed up to create pools of water. Cation analysis was performed on a PerkinElmer 5300 DV optical emission ICP with autosampler. Anion analysis was performed on-site using a HACH DR2010 spectrophotometer with protocols provided by the manufacturer. O and H isotopes were measured with a GV IsoPrime gas source mass spectrometer, with analytical precision of approximately 0.1 and 1 per mil, respectively.

### Scanning electron and confocal fluorescence microscopy

Biofilm samples for scanning electron microscopy were fixed for 2 h in a 2% glutaraldehyde solution (in 0.1-M sodium cacodylate buffer) according to a standard protocol, then vacuum aspirated onto 0.22-µm polycarbonate filters (Osmonics, poretics, 47 mm, catalog number K02CP04700), and rinsed three times in 0.1-M sodium cacodylate buffer. The samples were then dehydrated in successive ethanol baths of increasing concentration and dried using a Tousimis Autosamdri 815 Critical Point Dryer for approximately 1 h. Specimens were mounted on gold stubs and sputter coated with a gold/palladium mix. Imaging was performed on a Hitachi S-5000 scanning electron microscope at 10 keV at UC Berkeley.

Biofilm samples for confocal fluorescence microscopy were prepared with fluorescence dyes. Cell membranes and nucleic acids in the MS4 and MS11 biofilms were stained by adding simultaneously the lipophilic dye F4-64 (20 µg/mL) (Thermo Fisher, Grand Island, NY, USA) and SYTOX Blue (20 µg/mL) (Thermo Fisher, Grand Island, NY, USA), respectively. Samples were incubated at room temperature for 20 min, and then a 10-µL aliquot of biofilm was deposited onto a glass slide. Samples were imaged using a Leica Stellaris 5 confocal fluorescence microscope. Images were acquired using a 63 × oil immersion objective with laser excitation for SYTOX Blue (480 nm) and FM4-64 (520 nm), keeping the same parameters for both biofilms. Unmixed images were combined and false colored using the Leica Application Suite X (LAS X). All data was collected at the Innovative Genomics Institute, UC Berkeley.

### Scanning transmission X-ray microscopy (STXM)

STXM and near-edge X-ray absorption fine structure (NEXAFS) spectroscopy measurements were performed on the soft X-ray undulator beamline 11.0.2 [35] of the Advanced Light Source (ALS), Berkeley, CA, USA. Data were recorded with the storage ring operating in top-off mode at 500 mA, 1.9 GeV. Frozen samples were thawed right before STXM-NEXAFS measurements at ambient temperature under He at pressure < 1 atm. A Fresnel zone plate lens (40-nm outer zones) was used to focus a monochromatic soft X-ray beam onto the sample. The sample was raster scanned in 2D through the fixed beam, and transmitted photons were detected with a phosphor scintillator-photomultiplier assembly; incident photon counts were kept below 10 MHz. The imaging contrast relies on the excitation of core electrons by X-ray absorption [36–38]. STXM images recorded at energies just below and at the elemental absorption edge (S L_3_ and C K) were converted into optical density (OD) images where the OD for a given energy can be expressed from the Beer-Lambert law as *OD* =  − ln(I/I_0_) = µ *ρ* t, where I, *I*_0_, µ, *ρ*, and t are the transmitted intensity through the sample, incident intensity, mass absorption coefficient, density, and sample thickness, respectively. Protein, carbon, and elemental sulfur maps were obtained by taking the difference of OD images at 280 and 288.2 eV, at 280 and 305 eV, and at 162 and 163.9 eV, respectively. Image sequences (“stacks”) recorded at energies spanning the S L_2,3_ edges (160–180 eV) with steps of 0.3 eV around the L_3_-edge and C K-edge (280–305 eV) with steps of 0.12 eV around the K-edge were used to obtain NEXAFS spectra from specific regions. S L_2,3_ edges NEXAFS spectra are affected by spin–orbit coupling (multiplet structure) and provide information on the oxidation state of sulfur. Beam-induced radiation damage was carefully checked.

Additionally, STXM-NEXAFS measurements at 87 K were performed on frozen-hydrated samples deposited on Si_3_N_4_ windows so as to preserve sample morphology and chemical integrity [39] and minimize beam-induced radiation damage [40]. We made sure to analyze exclusively fully frozen-hydrated regions of the samples by first imaging the entire window. The samples were cryo-transferred through a specimen chamber (< 100 mTorr) into an LN_2_-cooled stage (87 K) inside the STXM operated with a scanning Fresnel zone plate lens (60-nm outer zones), under vacuum (10^−6^ Torr). With this setup, the sample is not rastered-scanned through the fixed beam so as to minimize sample vibrations; instead, the zone plate is scanned in 2D. Note that sulfur L_2,3_ edges could not be accessed with this setup due to geometrical constraints.

At least two different sample regions were analyzed at each elemental edge. The theoretical spectral and spatial resolutions during measurements were + / − 100 meV: 40 nm (at room temperature) and 60 nm (at 87 K) respectively. The photon energy was calibrated at the C K-edge using the Rydberg transition of gaseous CO_2_ at 292.74 eV (C 1 s → 3 s (*ν* = 0)). Sulfur spectra were calibrated using the S 2p_3/2_ edge of elemental sulfur set at 163.9 eV. An elemental sulfur standard spectrum was kindly provided by Geraldine Sarret [41] (Universite Grenoble Alpes). A lipid standard compound (PE) was kindly provided by Susan Glasaeur (University of Guelph). Carbon spectra were pre-edge background subtracted using a linear function and normalized at 300 eV. All data was processed with the aXis2000 software version 06 Jul 2021 (http://unicorn.mcmaster.ca/aXis2000.html). Sulfur spectra were further pre-edge background subtracted and post-edge normalized using Athena version 0.9.26 (Demeter package[42]).

### X-ray fluorescence microprobe (XFM)

Synchrotron XFM measurements were performed in cryogenic conditions (95 K) at ALS XFM beamline 10.3.2 [43], with the storage ring operating in top-off mode at 500 mA, 1.9 GeV. Micro-focused X-ray fluorescence (µXRF) elemental mapping was performed on flash-frozen hydrated samples oriented at 45° to the incident X-ray beam; frozen biofilm samples were mounted onto a TEM cartridge in a liquid nitrogen bath and cryo-transferred into a LN_2_-cooled apparatus following methods described elsewhere [44]. All data were recorded using a single-element XR-100 silicon drift detector (Amptek, Be window).

XRF maps were recorded at 4138 eV (100 eV above the Ca K-edge) using a beam spot size of 3 µm × 4 µm, 2 × 2 µm pixel size, and 70-ms dwell time/pixel. Micro-XRF spectra were recorded simultaneously on each pixel of the maps. All maps were then deadtime corrected and decontaminated using custom LabVIEW 2018 (National Instruments, Austin, TX, USA) software available at the beamline. Maps were then processed using a custom MATLAB R2020b program (MathWorks, Natick, MA, USA) available at the beamline.

### DNA extraction and metagenomic sequencing

Approximately, 200 µl of biofilm was extracted using MoBio PowerSoil DNA extraction kit (MoBio Laboratories, Inc., CA, USA) according to the manufacturer’s protocol, with the bead-beating time reduced to less than 1 min. This DNA extract was then gel purified and quantified using a low-mass ladder (Promega).

Total genomic DNA for metagenomic sequencing (150-bp or 250-bp reads) for both biofilm and planktonic samples (20% of each filter) was extracted using MoBio PowerMax Soil DNA extraction kit. Cells were extracted from 20% of each filter by adding 15 ml of lysis buffer and vortexing for 10 min. Lysis of cells was modified by heating to 65 °C for 30 min and 1 min of bead beating. DNA was eluted in Milli-Q water, and ethanol precipitation was performed (70% EtOH, 3-M sodium acetate, incubation for 24 h at 4 °C).

### Illumina sequencing, assembly, binning and sequence curation

Shotgun genomic reads were assembled using IDBA-UD [45]. Contigs larger than 2.5 kb were retained, and sequencing reads from all samples were mapped against each resulting assembly utilizing Bowtie 2 [46]. Differential coverage profiles, filtered with a 95% read identity threshold, were then used for genome binning using a suite of binning tools (MetaBAT2 [47], VAMB [48], MaxBin2 [49], Abawaca (https://github.com/CK7/abawaca), with the final bin choice determined by DAS Tool [50]. Draft genomes consisting of scaffolds ≥ 1 kbp in length were binned using ggKbase manual binning tools based on a combination of GC content, coverage, single-copy gene content, phylogenetic profile, and patterns of organism abundance over samples. The phylogenetic profile was established using representative genomes from the GTDB database. In some cases, scaffold sequences from groups of bins were used to construct emergent self-organizing maps in which the structure was established using tetranucleotide composition (tetra-ESOMs). For scaffolds > 6 kb, scaffolds were subdivided into 3-kb segments and treated separately in the ESOM analysis. In cases where the majority of segments from the same scaffold did not group together in the ESOM, the scaffolds were evaluated manually (based on gene content and other information) to resolve their placement or assign them to unbinned. The scaffold set defined based on ESOM analysis was then used to generate a draft genome bin that was again checked for consistent binning signals (as above). As ESOMs only used scaffolds > 3 kb in length, scaffolds from the original bins were added if they had a tightly defined GC, coverage, and the expected phylogenetic profile. CheckM2 [51] was used for estimation of genome completeness, strain heterogeneity, and contamination. Curated genomes with less than 5 duplicated single-copy genes (some of which occur because genes are split at scaffold ends) and with ≥ 95% of the expected single-copy marker gene were used for completeness estimation: at least 42 of 51 single-copy genes used for preliminary bin evaluation in ggKbase were classified as near complete. Genomes with > 5 duplicated single-copy genes were classified as partial, regardless of other indicators of bin completeness.

### Phylogenetic analyses

The concatenated ribosomal protein tree was generated using 16 syntenic genes that have been shown to undergo limited lateral gene transfer (rpL2, 3, 4, 5, 6, 14, 15, 16, 18, 22, 24 and rpS3, 8, 10, 17, 19) [52]. We obtained branch support with the ultrafast bootstrap [53] implemented in iQ-TREE v1.6.12 [54] with the following parameters: − bb 1000 − m LG + F + G4. Trees were visualized using iTOL v6.3.2 [55]. Amino acid alignments of the individual ribosomal proteins were generated using MAFFT v7.304 [56] and trimmed using trimAL [57] with the following setting: − gt 0.1.

To verify the presence of biogeochemically relevant genes, phylogenetic trees were constructed. We used gene markers for sulfur (DsrAB, Pdo), carbon metabolism (RuBisCO), and energy conservation ([NiFe]-hydrogenases). Sequences were obtained using GOOSOS and aligned using MAFFT v7.304. All other phylogenies were generated using iQ-TREE v.1.6.12 using the ultrafast bootstrap and parameters specified previously.

Hydrogenase sequences from Alum Rock genomes were obtained using HMMs [58]. The phylogenetic classification was performed using reference sequences obtained from [58] and HydDB [59]. Verification of hydrogenase loci was performed via inspection of nearby genes and the presence of required hydrogenase accessory genes. Genome context diagrams were generated using Clinker [60].

### Metagenomics metabolic pathways analysis

Preliminary functional annotations were established using METABOLIC [61], and collections of metabolic capacities in genome bins were overviewed using ggKbase tools [62]. In addition, metabolic profiling was done by mapping ORFs to KEGG ortholog groups (KOs) using an HMM database that was compiled as previously described [63]. This HMM database was used to scan the metagenomic bins, and ORFs were assigned the KO of the best-scoring HMM, providing it was above the noise threshold. In addition, we profiled metabolic capacities with KEGG functional annotation using METABOLIC [61].

### Protein structure prediction

Protein structures were predicted for the putative complexes of the nitrate reductase (Nrx), dioxygenase/rhodonase, and group 3b [NiFe]-hydrogenase using AlphaFold2 in multimer mode. Specifically, for the 3b [NiFe]-hydrogenase complexes, AlphaFold2 was used in multimer mode for the HyhL (hydrogenase large subunit), HyhS (hydrogenase small subunit), HyhG (diaphorase catalytic subunit), and HyhB (diaphorase electron transfer subunit) [64, 65]. In all cases, the average per residue confidence scores (pLDDT) exceeded 90, a level that is empirically shown to produce highly accurate local structural models. The best-scoring models were aligned to related protein complexes in PyMol.

## Results

### Groundwater of mixed origin hosts biofilms dominated by filamentous bacteria

We measured the flow rate, pH, and concentrations of ionic species (Supplementary Table S1) in the MS4 and MS11 groundwater. The MS11 spring has a higher flow rate, ionic strength, alkalinity, and sulfide levels than the MS4 spring. H and O stable isotope compositions of the waters, combined with salinity measurements, indicate that spring waters are mixtures of meteoric input and pore waters from the host Miocene Monterey Group shales and cherts and possibly deeper Cretaceous sediments of the Great Valley Group. MS4 water is more diluted by meteoric input than MS11. Long-term monitoring of these two springs shows they experience small seasonal fluctuations in temperature, and that they are generally hydrologically and geochemically stable (Fig. 1D–F). Water temperatures of 27–29 °C are well above the mean annual surface temperature of 15.1 °C. The salinity of the springs is 1.8 and 2.3% for MS4 and MS11, respectively. The sulfide levels (within the zone of oxygenation) range up to ~ 9 and 69 μmol/L at MS4 and MS11, respectively.

**Fig. 2:**
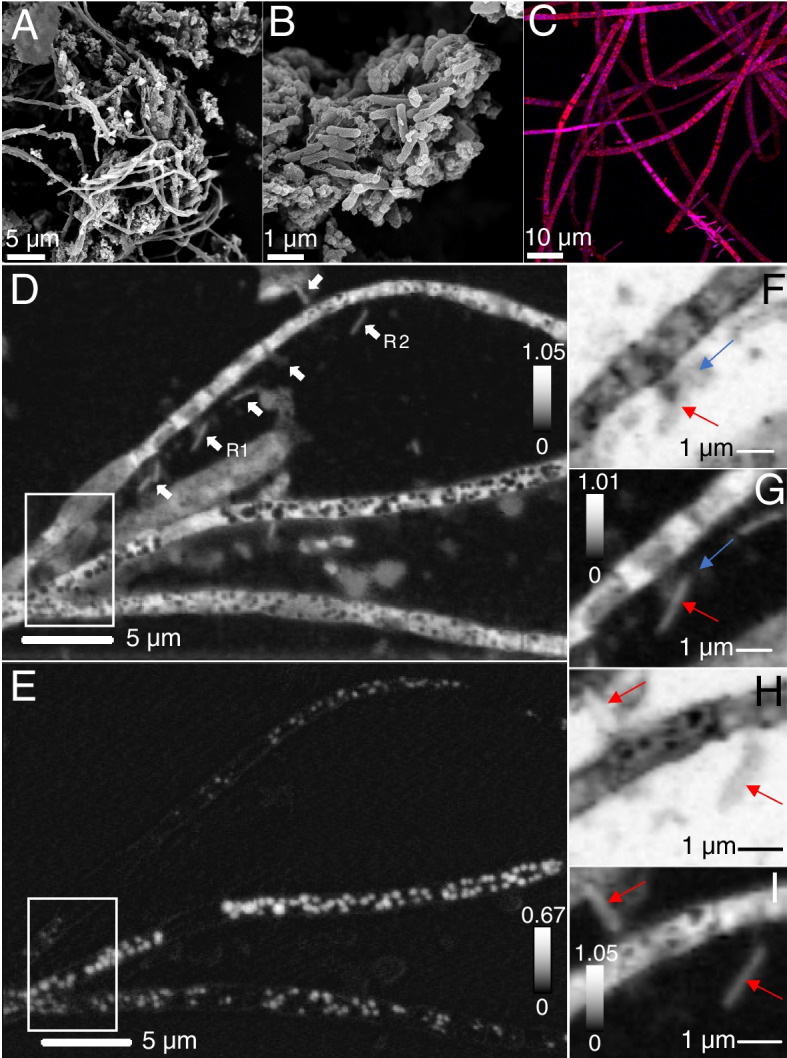
Microscopic characterization of MS4 biofilms. **A**–**B** Scanning electron microscopy of filamentous bacteria and associated cells, small cells are pointed by arrows (**C**) confocal fluorescence microscopy of cells treated with SYTOX (blue) for nucleic acid and F-64 (red) for membrane. Scanning transmission x-ray microscopy: (**D**) carbon map of filaments and associated cells (white arrows). **E** Corresponding distribution map of S^0^ evidencing spherical elemental sulfur granules within the compartments of the filaments. The top, middle, and bottom filament widths are 1.23 ± 0.5 µm, 1.01 ± 0.2 µm, and 1.33 ± 0.3 µm, respectively. **F** An ultra-small cell ~ 480 nm long, ~ 270 nm wide, (blue arrow) in contact with an apparently episymbiotic cell 1.86 ± 0.1 µm long, ~ 360 nm wide (red arrow), imaged at 280 eV (region R1, **D**) and corresponding (**G**) carbon map. **H** Two apparently episymbiotic cells (red arrows) connected to filaments, imaged at 280 eV (region R2, **D**) and corresponding (**I**) carbon map. The intensity scales correspond to optical density

The biofilms at both MS4 and MS11 sites (Fig. 2A, B) are mainly composed of thin white streamers (~ 5–10 cm long) that are primarily attached to rocks. Scanning electron microscopy (SEM), confocal fluorescence microscopy (CFM), and scanning transmission X-ray microscopy (STXM) revealed that MS4 biofilms consist of filaments and cells distributed among the filaments (Fig. 2), whereas the MS11 biofilm consists mainly of filamentous bacteria (Fig. S2).

### Filamentous bacteria have encapsulated elemental sulfur granules and episymbionts

**Fig. 3:**
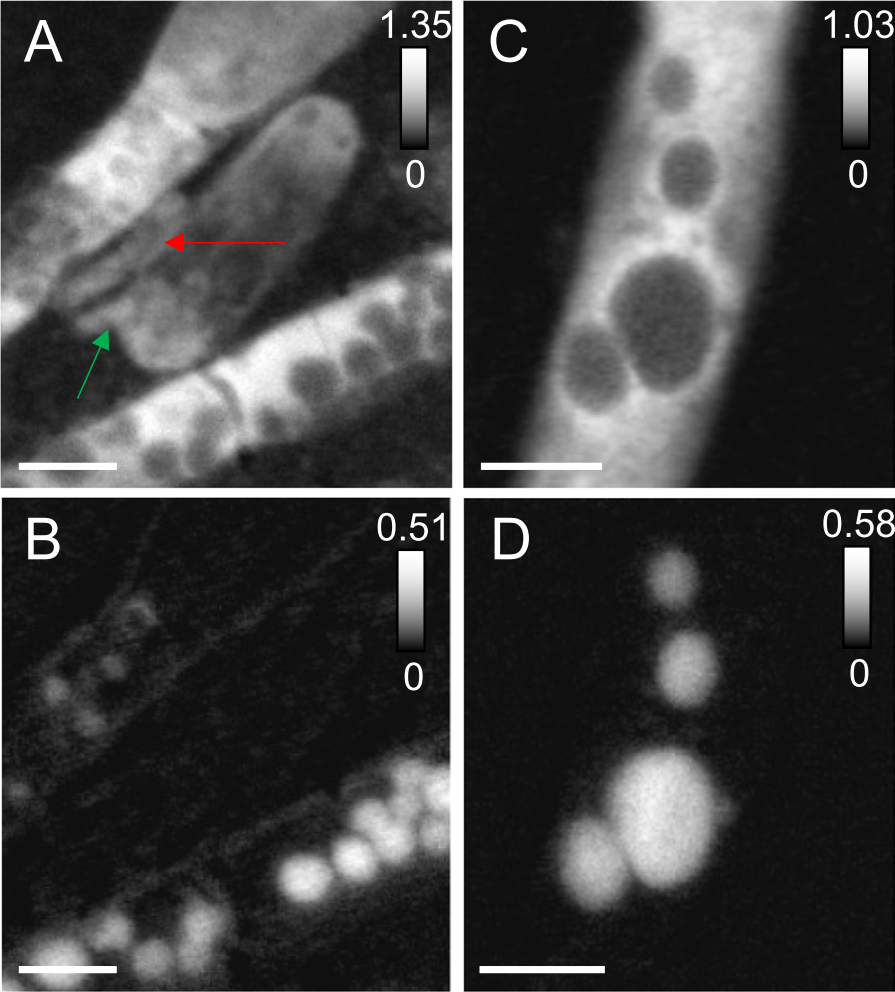
Scanning transmission X-ray microscopy of MS4 and MS11 biofilms. **A** Protein map and corresponding (**B**) distribution map of S^0^ in MS4 biofilms (in white boxed area of Fig. 2). Cells that are 908 ± 32 nm long, 370 ± 30 nm wide (red arrow), 687 ± 34 nm long, 244 ± 33 nm wide (green arrow), seen in close contact with filaments. **C** Protein map and corresponding (**D**) distribution map of S^0^ in MS11 biofilms, showing the presence of sulfur granules (up to 1.08 ± 0.12 µm in diameter) in a small area of a long filament. Sulfur L_2,3_ edge spectra of the granules can be found in Fig. S2. The intensity scale corresponds to the optical density. Scale bars: 1 µm

**Fig. 4:**
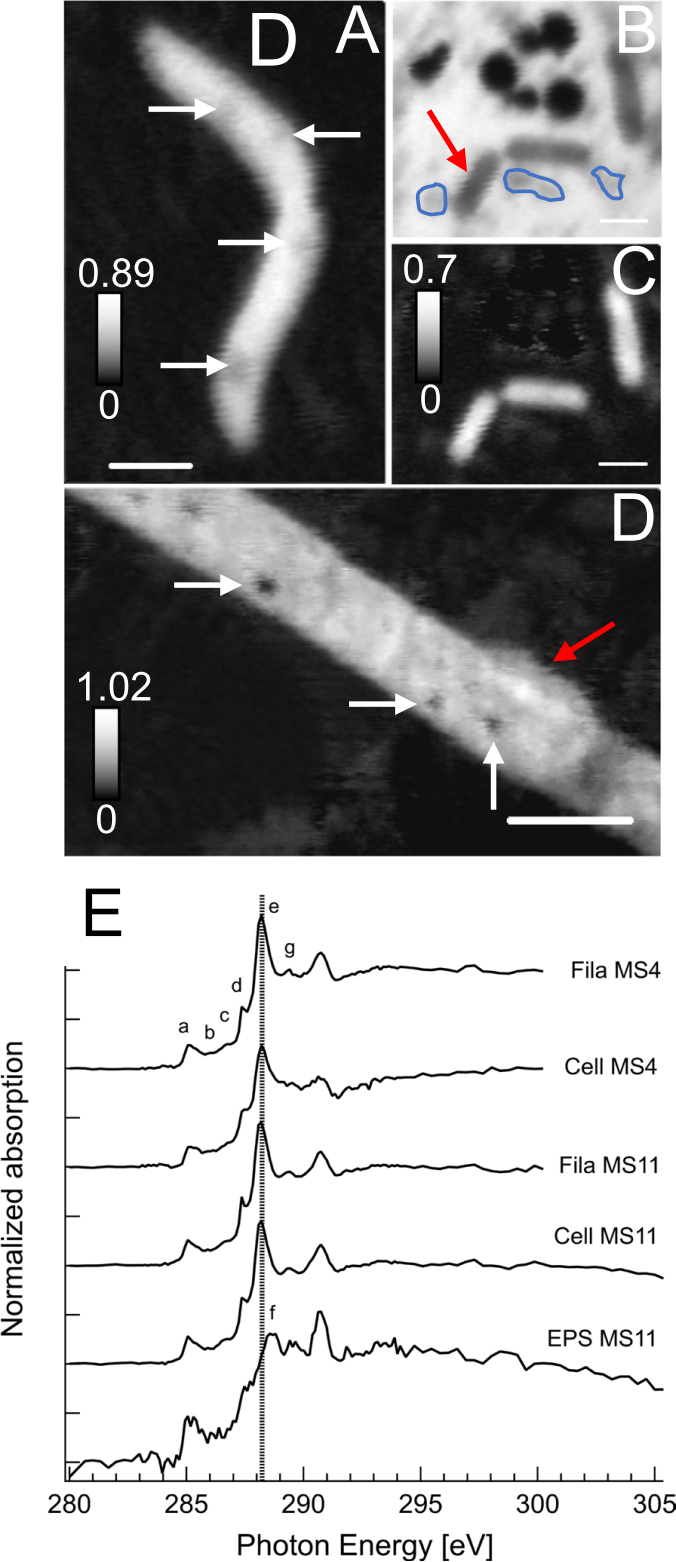
Scanning transmission x-ray spectromicroscopy at 87 K of frozen-hydrated MS4 and MS11 biofilms. **A** Protein map of an MS11 small filament (700 nm ± 120 nm in diameter), S^0^ granules are pointed by white arrows. **B** Extracellular S^0^ granules (~ 300 to 850 nm in diameter) and MS11 cells imaged at 288.2 eV (amide group in proteins) and corresponding (**C**) protein map. **D** Protein map of an MS4 filament (1.46 ± 0.16 µm in diameter) with a surface-attached cell (1.9 ± 0.21 µm, red arrow). **E** Carbon K-edge NEXAFS spectra of filamentous bacteria (S.^0^ granule-free areas) exhibiting a major peak at 288.2 eV and other peaks mainly associated with nucleic acids (see Table S2). Spectra of MS11 and MS4 cells (red arrow) exhibit a main peak at 288.2 eV (peptide bond), and associated extracellular polymeric substances (EPS, circled in blue) show a main peak at 288.7 eV (carboxyl groups in acidic polysaccharides). See Table S2 for further details. Dashed line is at 288.2 eV. The intensity scale corresponds to the optical density. Scale bars: 1 µm (**A**–**C**) and 2 µm (**D**)

Sulfur μXRF distribution maps at 95 K evidenced the presence of sulfur across MS4 filamentous bacteria (Fig. S1). STXM sulfur maps (Fig. 2) and S L_2,3_ NEXAFS spectra showed that the filaments contain spherical S^0^ granules (Fig. S3) encapsulated in protein-rich compartments (Fig. 3A, B). The spherical granules in the middle filament (Fig. 2E) are roughly 380-nm average diameter, as estimated from 76 granules. The width of these MS4 filaments is < 1.6 µm. Filaments exhibit septa and longitudinal cell envelopes as observed by STXM (Fig. S4) and CFM (Fig. 2). Rod-shaped, curved-shaped, and few coccoid-shaped cells were found near the filaments in MS4 biofilms, as well as ultra-small cells (Fig. 2, Fig. S4D), often found within extracellular polymeric substances (EPS). Higher-resolution protein maps of MS4 and MS11 filaments (Fig. 3) suggest that sulfur granules are surrounded by proteins, as further confirmed on maps recorded at 87 K (Fig. 4).

Carbon K-edge NEXAFS spectra at 87 K of filamentous bacteria in MS4 and MS11 biofilms (Fig. 4) exhibit similar spectra (Supplementary Table S2), with a major peak at 288.2 eV (amide carbonyl groups in proteins [66]), a peak at 285.2 eV attributed mainly to aromatic groups in proteins, and peaks at 286, 286.6, 287.4, and 289.4 eV that can be attributed to nucleic acids [67–69] (Fig. S5). One prior report [69] suggests that spectra of nucleotide bases with peaks occurring within 284.7 − 286.9 eV are likely associated with π*(C = C), whereas peaks in the 287.3–287.4 eV range are likely associated with π*(C = N). Resonances are more defined than in prior studies at room temperature [37, 44, 70, 71], due to reduced Debye–Waller thermal disorder at low temperatures. This in turn allows us to unequivocally detect the presence of nucleic acids in the filaments, in addition to proteins. Copious extracellular polymeric substances surrounding MS4 and MS11 filaments exhibit a main peak at 288.7 eV, which can be attributed to carboxyl groups in acidic polysaccharides (Supplementary Table S2). The spectra of MS11 cells are also similar to those of filamentous bacteria, with a major peak at 288.2 eV (amide bonds) and other peaks associated with nucleic acids. The spectrum of a surface-attached cell on an MS4 filament exhibits a major peak at 288.2 eV (amide bonds) and nucleic acid-associated peaks; the 288.2 and 285.2 eV peaks are broader likely due to the presence of EPS surrounding this cell. Cells, filaments, and EPS all exhibited a shifted carbonate peak at 290.7 eV that corresponds to either organic carbonates or carbonate minerals [72] and originates mainly from dissolved carbonates and carbonate precipitates present in the groundwater at circumneutral pH (Supplementary Table S1 and S2)

Strikingly, small cells were found along the surfaces of the filaments in MS4 (Fig. 2F, G, Fig. S4) and MS11 (Fig. S4A) biofilms; these cells are typically about 480 nm long, 250 nm wide, as estimated from STXM images. Ultra-small cells (290 ± 20 nm long, 120 ± 15 nm wide) were also found in close proximity to the filaments in MS4 (Fig. S4D).

### Biofilms contain diverse bacteria including CPR bacteria

To determine the bacterial community composition across biofilm samples collected at MS4 and MS11 sites over the years, we analyzed the relative abundance of bacterial taxa at the phylum, class, and order levels. We observed a diverse range of bacterial phyla across the samples (Fig. 1G, see also supplementary material for details). The most abundant phylum across the samples was Campylobacterota, representing > 50% of the total bacteria, followed by Proteobacteria and Desulfobacterota, respectively. Data on the MS11 biofilms in the year 2020 indicate a possible shift in the bacterial community structure during this period. A closer look at the class level revealed that Gammaproteobacteria are predominant in most samples from the MS4 site, representing a significant portion of the bacterial community. However, distinct patterns of class-level diversity were evident in different samples, showcasing the dynamic nature of biofilm communities over the years at both sites.

**Fig. 5:**
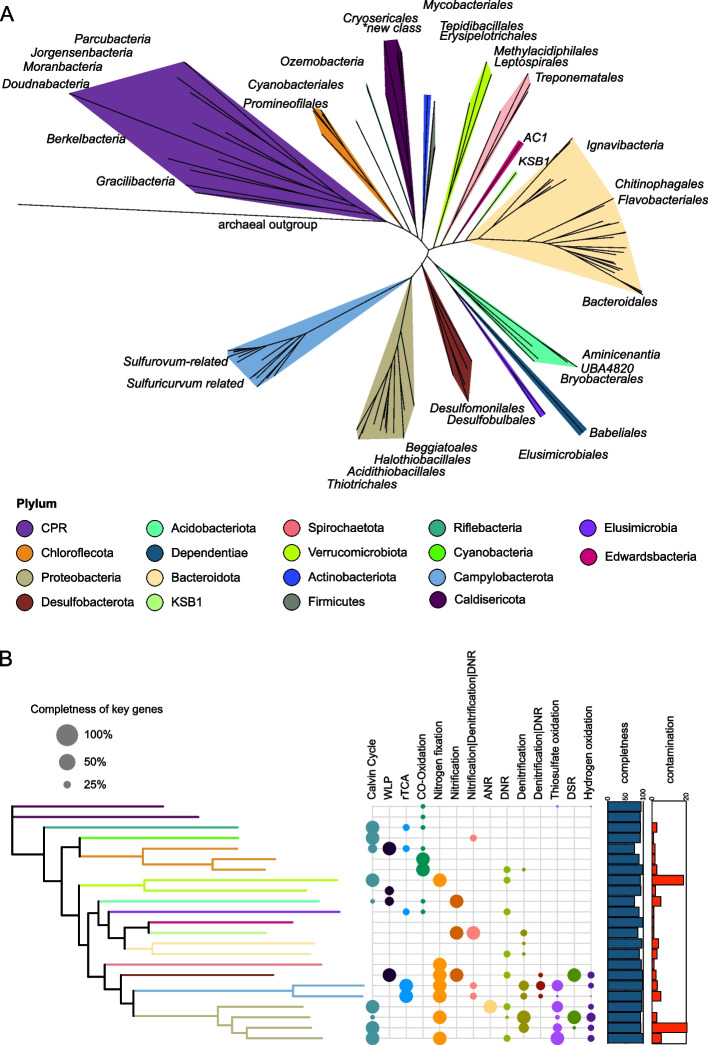
Phylogenetic analysis and metabolism of bacteria represented by MAGs from the MS4 and MS11 sites. **A** The tree is based on 16 concatenated ribosomal proteins (rpL2, 3, 4, 5, 6, 14, 15, 16, 18, 22, 24, and rpS3, 8, 10, 17, 19) generated using iQ-TREE. An archaeon, *Thermococcus alcaliphilu*s, was used as the outgroup. **B** The metabolic capacities for generalized biogeochemical pathways in Alum Rock genomes are represented by colored circles. A pathway is present if the core KEGG orthologs encoding that pathway are identified in each genome. Abbreviations are as follows: WLP, Wood–Ljungdahl pathway, rTCA, reductive tricarboxylic acid cycle; ANR, assimilatory nitrate reduction; DNRA, dissimilatory nitrate reduction to ammonia; thiosulfate oxidation by SOX complex; DSR, dissimilatory sulfate reduction; hydrogen oxidation, [NiFe]-hydrogenases, and NAD-reducing hydrogenase

We used genome-resolved metagenomics to investigate microbial consortia, metabolisms, and microbial interactions that underpin the Alum Rock communities. In total, we recovered 212 nonredundant genomic bins from the MS4 and MS11 samples (57 from MS11 and 155 from the biofilm + planktonic samples from MS4). Of these, 38 were classified as near complete (> 95%, Supplementary Table S3). Taxonomic affiliations of all of the bacterial genomes were established based on concatenated ribosomal protein trees (Fig. 5A and Supplementary Table S4).

Genomically represented groups in the biofilms and planktonic fractions from both sites include Gammaproteobacteria (Thiotrichales, Chromatiales, Beggiotales), Campylobacterota (Campylobacterales), Betaproteobacteria (including *Thiomonas*), Deltaproteobacteria (specifically Desulfobacterales), Bacteroidota, Chloroflexota, Ignavibacteria, Spirochaetes, Lentisphaerae, Riflebacteria, Verucomicrobia, Acidobacteria, KSB1, Caldisericota, Planctomycetota, Edwardsbacteria, Dependentiae (TM6), and Margulisbacteria. Diverse groups of CPR are present, including Uhrbacteria (OP11), Gracilibacteria (BD1-5), Peregrinibacteria (PER), Moranbacteria (OD1), Woesebacteria (OP11), Roizmanbacteria and Gottesmanbacteria (OP11), Saccharibacteria (TM7), Falkowbacteria (OD1), Absconditabacteria (SR1), Berkelbacteria, and Doudnabacteria and Dojkabacteria (WS6) (see https://ggkbase.berkeley.edu/alumrock-genomes/organisms).

To estimate the relative abundance of organisms in the two springs (independent of binning), we calculated the DNA read coverage of all of the genomic bins for each spring (Fig. S6). The MS4 spring is dominated by Halothiobacillales, Beggiatoales, Thiotrichales, and Campylobacterales based on relative abundance among genomes (Supplementary Table S5). The most abundant species in MS4 shares genome-wide average 51% amino acid similarity with the sulfur-oxidizer *Thiothrix nivea* [73]. The MS11 spring is dominated by a single *Beggiatoa* sp. (Beggiatoa-related_37_1401).

### Diverse bacteria are implicated in sulfur cycling

We used a list of high-confidence functional gene assignments for all draft or better-quality genomes to resolve the capabilities central to the metabolisms of the dominant bacteria in the springs. Not surprisingly, genes encoding sulfur cycling are common in the most abundant organisms at both sites (Supplementary Table S5A, B).

Based on the community composition of MS4 and MS11 biofilms, we further focused our analysis on the metabolic pathways of MS4 bacteria where we detected ultra-small and surface-attached cells on filamentous bacteria implicated in sulfur oxidation. The most abundant organism in MS4, which is closely related to the filamentous bacterium *Thiothrix nivea*, encodes genes (*soxABC*, periplasmic thiosulfate-oxidizing; *aprAB*, adenylylsulfate reductase; *dsrAB*, reverse dissimilatory sulfite reductase) to convert sulfide to thiosulfate, elemental sulfur, and sulfate (Fig. 5B). The absence of *dsrD* genes indicates that the Dsr complex operates in the sulfide oxidation direction (i.e., rDsr pathway). This *Thiothrix* bacterium also lacks any *soxC* genes, which in bacterial genomes has been associated with the accumulation of sulfur granules or polysulfide [74, 75].

MS4 contains various other bacteria capable of oxidizing reduced sulfur compounds. A subdominant population of *Sulfurovum* bacteria encode *sqr* genes and thus likely oxidize sulfide to S^0^. Some *Sulfurovum* bacteria in both communities have genomes that also encode *soxCDYZ* complexes, suggesting they mediate thiosulfate oxidation (potentially coupled to nitrate reduction, e.g., via *narG* and *napA*). *Sulfuricurvum* species are also relatively abundant in MS4 and encode genes for sulfur and thiosulfate oxidation, in line with culture-based studies [76]. The genomes of Chloroflexota encode the capacity for thiosulfate disproportionation via thiosulfate reductase/polysulfide reductase (*phsA*) and sulfide oxidation via flavocytochrome *c* sulfide dehydrogenase. Two low abundance Gammaproteobacteria species related to *Acidithiobacillus* have the capacity for thiosulfate oxidation. Several genomes from moderately abundant Halothiobacillales have the metabolic capacity for sulfide and thiosulfate oxidation via *fccB*, *dsrAB*, and *soxBCY*, respectively (Supplementary Table S6A and B).

Some bacteria from MS4 spring also potentially mediate dissimilatory sulfate reduction. Specifically, the genomes of some Desulfobacterales belonging to the families of Desulfatiglandaceae, Syntrophobacterales, Desulfurivibrionaceae, and Desulfarculales encode the capacity to reduce sulfate back to sulfide via Dsr genes, likely coupled to oxidation of organic carbon or H_2_. Some rare Desulfocapsaceae from MS4 that are related to bacteria of the genus *Desulfocapsa* have thiosulfate reductase, group 3b [NiFe] (Hyd; possibly sulfhydrogenase), and SAT (Sulfate adenylyltransferase) and APR (adenylylsulfate reductase) for the oxidation of sulfite to sulfate. Thus, it appears these bacteria are involved in sulfur disproportionation, whereby S^0^, thiosulfate, and sulfite are converted to H_2_S and sulfate, as has been demonstrated in cultures of bacteria from this genus [77]. Other *Desulfocapsa* spp. have tetrathionate reductase genes, suggesting they are capable of converting tetrathionate to thiosulfate. The *Desulfocapsa*-related bacteria also contain *dsrABD* genes, which fall within the reductive cluster closely related to those from *Desulfocapsa sulfexigens*. We infer that the *Desulfocapsa*-related bacteria are capable of S disproportionation, as previously reported [78], and the presence of the *dsrD* functional marker protein suggests that these species in the springs are capable of both S disproportionation and sulfite reduction. Only members of the candidate phylum Riflebacteria, family Ozemobacteraceae, have the capacity of anaerobic sulfite reduction via anaerobic sulfite reductase system (*asrABC*). A bacterium from a new class of *Caldithrix* from the MS4 spring is predicted to perform sulfur oxidation via dissimilatory sulfite reductase, sulfite oxidation, sulfate reduction, and thiosulfate disproportionation (Supplementary Table S6A). We also identified abundant bacteria from novel families of Bacteroidetes, which generally encode thiosulfate reductase genes (*phS*) and adenylylsulfate reductase (*aprA*) involved in thiosulfate disproportionation and sulfate reduction.

**Fig. 6:**
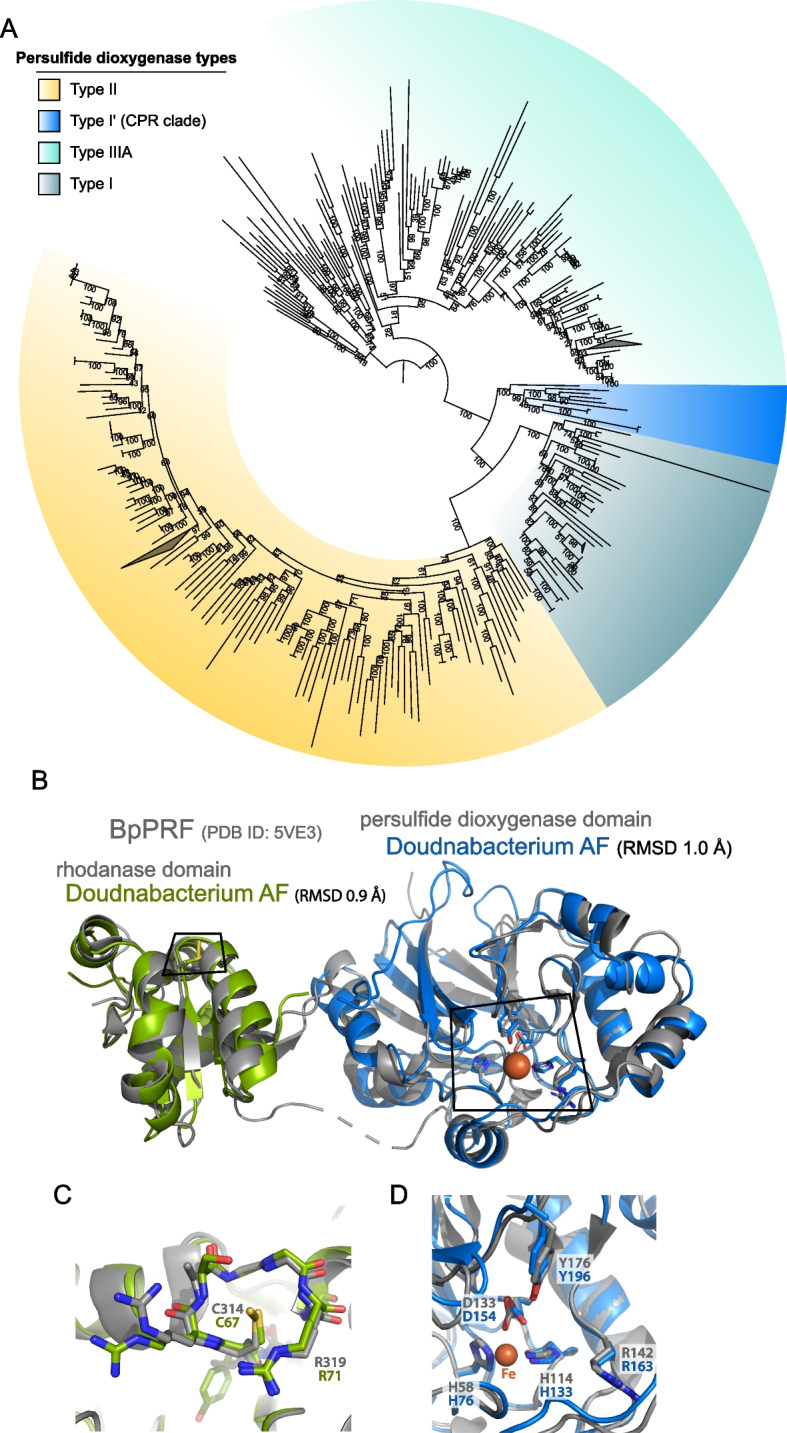
Novel persulfide dioxygenase within CPR bacteria. **A** Phylogenetic analyses of persulfide dioxygenase proteins from the Alum Rock genomic bins. The blue monophyletic clade shows the persulfide dioxygenase found in CPR bacteria from sulfur-rich environments. **B** AlphaFold models of Doudnabacterium putative rhodonase (green) and persulfide dioxygenase (blue) aligned with the corresponding domains of the characterized natural fusion protein BpRF (PDB ID: 5VE3). **C** and **D** Zoomed views of the active sites of the aligned structures reveal a strong coincidence of the key residues

Surprisingly, we identified persulfide dioxygenase (sdo) and rhodonase (thiosulfate sulfurtransferase) in the genomes of Elusimicrobia, Riflebacteria, and Oscillatoriophycidae and in a novel family of Syntrophales (Fig. 6A). These enzymes are also present in some heterotrophic bacteria, where they play important roles in the detoxification of intracellular sulfide and sulfur assimilation respectively [79, 80]. We also found a putative sulfur dioxygenase encoded in a Doudnabacteria genome that clusters with protein sequences of other CPR bacteria from public data. In the operon, a sulfur transferase is adjacent, suggesting its potential function in thiosulfate oxidation (Fig. 8). This is interesting because persulfide dioxygenase has not been previously linked to CPR bacteria. Modeling of the persulfide dioxygenase from Doudnabacterium using AlphaFold2 indicates that it has structural homology with the biochemically characterized persulfide dioxygenase (Fig. 6B–D). Furthermore, we identified these two adjacent genes in the genomes of several other CPR from high sulfide environments, including Kaiserbacteria (groundwater from California), Pacebacteria (wastewater), Moranbacteria, and Gracilibacteria (Crystal Geyser aquifer). Thus, we suggest that these genes may enable a variety of CPR bacteria to grow and generate energy from sulfur oxidation.

In contrast, the most highly sampled genomes in MS11 spring are from a *Beggiatoa* species including a novel family within this group (Supplementary Tables S6A). As expected, these *Beggiatoa* genome encodes the Dsr genes (*dsrABCHJKMOPR)*; *dsrD* genes were not identified, and we conclude that the Dsr genes are operational in a reverse Dsr pathway (rDsrABs) [81]. The genome also encodes AprAB (adenylylsulfate reductases) and Sat (sulfate adenylyltransferase) for the oxidation of sulfide to sulfate, sulfide-quinone oxidoreductase (Sqr), and sulfide dehydrogenase (*fccB*) genes for the oxidation of hydrogen sulfide to S^0^. The genomes contain a partial set of sulfur-oxidizing sox pathway genes, but *soxDXYZ* were identified. Given the lack of *soxC*, we conclude that like *Thiothrix*, the primary role of *Beggiatoa* in the community is the conversion of sulfide to thiosulfate, elemental sulfur, and sulfate. The absence of *soxCD* in bacterial genomes has been previously associated with the accumulation of sulfur granules or polysulfide [74, 75].

### Sulfur-oxidizing bacteria also contribute to nitrogen cycling

The dominant bacteria in MS4 and MS11 springs are predicted to mediate nitrogen fixation and denitrification processes. In both MS4 and MS11, genes encoding nitrogenase implicated in N_2_ fixation are widespread in Proteobacteria, including in the dominant *Thiothrix*, *Beggiatoa*, and *Sulfurovum*, Verrucomicrobia. Other organisms with this capacity include other Gammaproteobacteria, Chromatiales, Campylobacteriales, *Sulfuricurvum*, Ignavibacteria, *Sulfurospirillum*, Spirochaetes, *Desulfocapsa*, and potentially Lentisphaerae.

The Thiotrichales genomes encode numerous genes for the reduction of nitrate and nitrite; however, only the dominant *Thiothrix* species have the capability to reduce nitrite to nitrous oxide via *nirS* and *norBC* genes. Some Chromatiales bacteria at both springs also appear to be capable of dissimilatory nitrite oxidation to ammonia. The sulfur-oxidizing Campylobacterales that we identified in both MS4 and MS11 springs have numerous genes implicated in the reduction of nitrate (*napAB*) and nitric oxide (*norBC*). Two low abundance Acidithiobacillales in MS4 that are predicted to perform thiosulfate oxidation have ammonia monooxygenase (*amoA*) genes, suggesting they may be involved in ammonia oxidation and nitrite ammonification. Chloroflexota populations at both springs have the capacity for nitrite reduction via nitrite reductase (*nirK*), nitric oxide reduction (*norBC*), and nitrite ammonification. A novel *Caldithrix* species from MS4 has the potential of nitric oxide reduction via the nitric oxide reductase *norBC* and nitrite reduction via the periplasmic nitrate reductase NapA (Fig. 5B).

In addition to being the most abundant sulfur oxidizers in the MS11 spring, *Beggiatoa* are metabolically versatile with regard to nitrogen cycling. Their genomes encode genes with similarity to nitrate reductase (*narABG*), nitrite reductase (*nirS*), nitric oxide reductase (*norBC*), and nitrous-oxide reductase (*nosZ*) for the complete reduction of nitrate to N_2_. They also contain *nrfA* potentially for dissimilatory nitrite reduction to ammonia (DNRA) or nitrite ammonification. Thus, these bacteria can likely couple sulfur oxidation to nitrate reduction, in line with prior studies [3, 12].

### Extensive links between hydrogen and sulfur metabolism

**Fig. 7:**
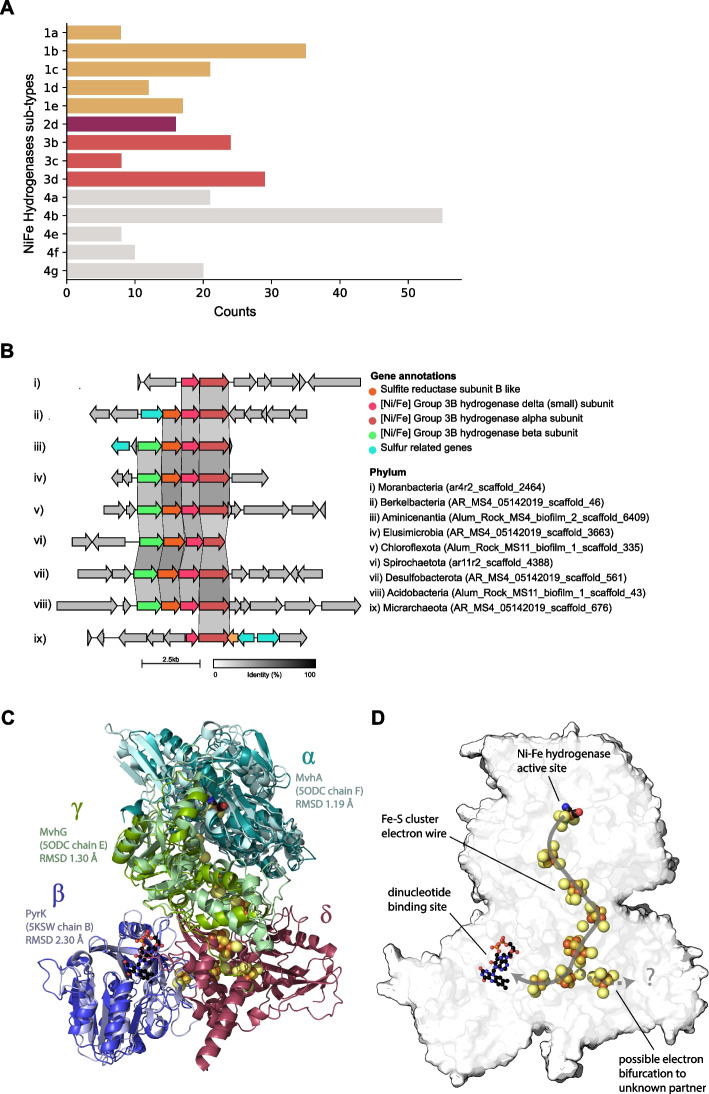
Hydrogenases distribution in Alum Rock genomes and structural insights of group 3b [NiFe]-hydrogenase complex. **A** Total distribution of hydrogenases from the Alum Rock spring. **B** Genomic organization of novel group 3b [NiFe] hydrogenases from different organisms present in the springs. **C** and **D** AlphaFold multimeric model for the Berkelbacterium putative group 3b [NiFe]-hydrogenase complex with the closest known structural matches aligned to each protein

To gain insight into the role of hydrogen metabolism in the Alum Rock springs, we analyzed the distribution of hydrogenases and associated enzymes in the genomes. There was considerable capacity for fermentative H_2_ production using nicotinamides (via group 3b and 3d [NiFe]-hydrogenases), ferredoxin (via group A [FeFe]-hydrogenases and group 4 [NiFe]-hydrogenases), and formate (via formate hydrogenases) as electron donors (Fig. 7A). Some putative H_2_ producers are likely to be metabolically flexible bacteria such as *Sulfurospirillum* and Flavobacteriales, which can switch to fermentation when limited for respiratory electron acceptors based on previous reports [59, 82]. CPR bacteria, TA06, and Spirochaetes with group 3b and 3d [NiFe]-hydrogenases are likely to be obligate fermenters given they apparently lack terminal reductases (Supplementary Table S7). The gene arrangements of the group 3b [NiFe]-hydrogenases in the genomes of the CPR bacteria Berkelbacteria and Moranbacteria (Fig. 7B) are similar to the biochemically characterized hydrogenase and sulfhydrogenase of *Pyrococcus furiosus* [83] and those previously reported in other CPR bacteria [25, 84], suggesting that these hydrogenases may be capable of reversible oxidation of hydrogen or capable of reducing sulfur compounds like polysulfide. We modeled the complex from Berkelbacteria genome using AlphaFold; this model suggests a hydrogenase module (*α*- and *γ*-subunits) with an electron wire of FeS clusters connecting to a nucleotide reducing module (β subunit) (Fig. 7C). The δ subunit has no close structural analogues but contains an additional FeS cluster and may accommodate an additional electron-accepting partner (Fig. 7D). Based on this structural analysis, there are two separate paths for the electrons suggesting this 3b [NiFe]-hydrogenase complex is potentially an electron-bifurcating hydrogenase.

Numerous bacteria in the Alum Rock springs are predicted to consume H_2_ for energy generation. Most of these hydrogenotrophs are predicted to use H_2_ to reduce sulfate (via group 1b and 1c [NiFe]-hydrogenases; primarily Deltaproteobacteria), elemental sulfur (via group 1e [NiFe]-hydrogenases; primarily Gammaproteobacteria), or heterodisulfides (via group 3c [NiFe]-hydrogenases; various lineages including Acidobacteria). The most abundant Gammaproteobacteria and Campylobacteria likely oxidize both H_2_ and sulfur compounds either mixotrophically or alternatively autotrophically. The hydrogenase repertoire of these organisms includes the oxygen-tolerant group 1b and 1d [NiFe]-hydrogenases [85, 86].

### Organic carbon cycling and fermentation

The ability to fix inorganic carbon (CO_2_) is a common predicted capacity for bacteria from both sites (Supplementary Table S6A and B). The dominant *Thiothrix*, *Beggiatoa*, and Chromatiales-related bacteria have type II RuBisCO genes that function in the Calvin-Benson-Bassham (CBB) cycle (Fig. S7). One Absconditabacteria genome has a RuBisCO that phylogenetic analysis places within the form II/III CPR clade, as reported previously [25, 87]; these enzymes are inferred to function in a nucleoside salvage pathway in which CO_2_ is added to ribulose-1,5-bisphosphate to form 3-phosphoglycerate [88]. Elusimicrobia and Campylobacterota, including species related to Sulfurimonadaceae, have ATP citrate lyase genes that encode the critical enzyme for CO_2_ fixation via the reverse TCA (rTCA) cycle. We also identified rTCA genes in a novel Bacteroidetes organism (Supplementary Table S6A and B). Genes of the Wood–Ljungdahl carbon fixation pathway (*cooS/acsA*, *acsB*, and *acsE*) were widespread in both springs, including in members of the Bacteroidetes, Desulfocapsa, Lentisphaerae, Chloroflexi, and Aminicenantia with the potential of oxidation of small organic compounds.

**Fig. 8:**
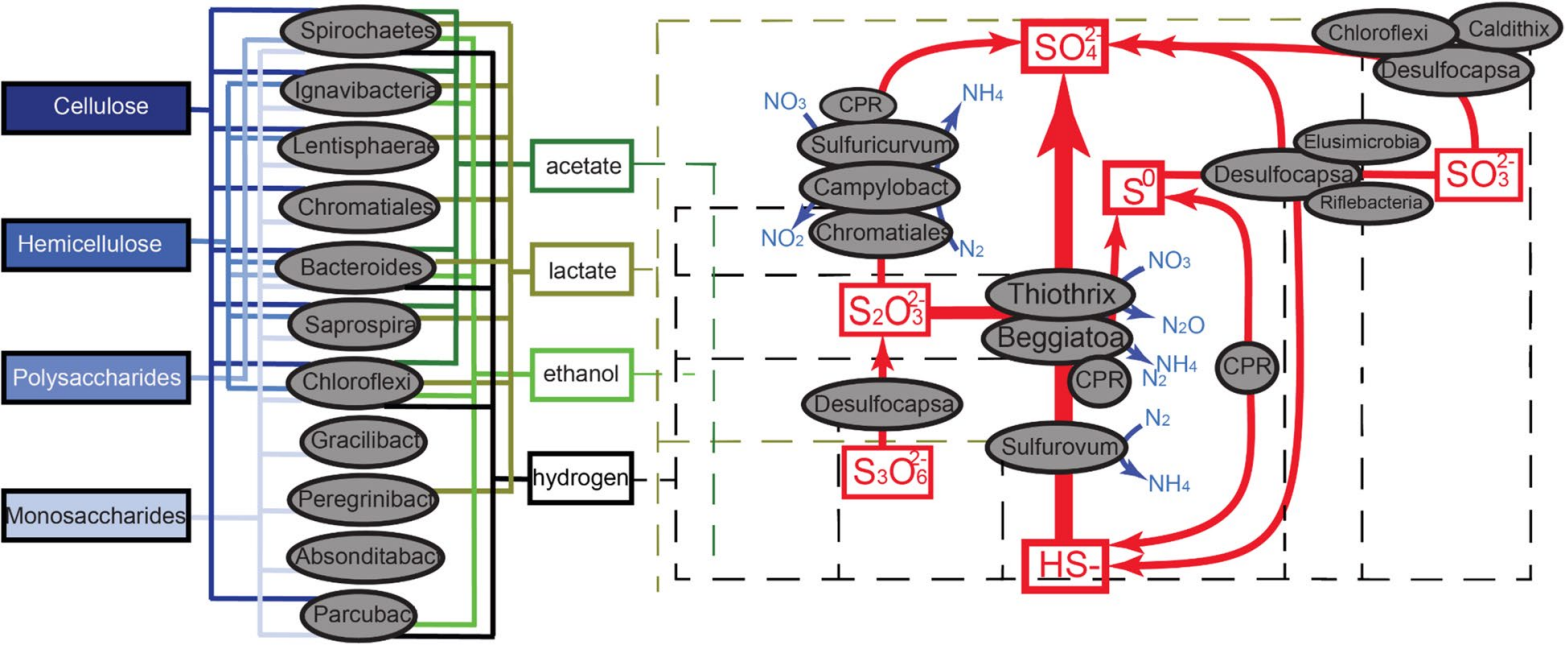
Inference of partitioning of carbon, sulfur, and nitrogen cycling in the Alum Rock springs. Based on the gene content of genomes reconstructed from the springs. Arrows indicate metabolic capacities reconstructed from metagenomes recovered from MS4 and MS11 springs. The dashed lines represent potential electron donors for anaerobic respiration processes

We used marker genes involved in carbohydrate metabolism to infer polymer biomass degradation capacity of the microbial communities. Many bacteria in both springs have the capacity to hydrolyze complex organic molecules to produce a variety of electron donors such as acetate, hydrogen, and lactate (Fig. 8A). Of the organisms in the community, Bacteroidetes and Ignavibacteria contain the most glycosyl-hydrolase genes, and thus, they likely play important roles in polysaccharide degradation. Notably, one Bacteroidetes from MS11 has 66 glycoside hydrolase genes. This organism is the only bacterium that appears to be capable of degrading cellulose, hemicellulose, polysaccharides, and monosaccharides. Gammaproteobacteria, Spirochaetes, Bacilli, and Lentisphaerae also contain genes for the degradation of a variety of complex carbohydrates, but these genes are at relatively low abundance in the sulfur-oxidizing Proteobacteria.

Similarly, many bacteria other than the sulfur-oxidizing Proteobacteria (and CPR) have indications of the capacity for beta-oxidation pathway of saturated fatty acids to acetyl-CoA. Many CPR bacteria have a few glycosyl hydrolase genes, which is significant given the scarce indications of other metabolic capacities in these microorganisms. Methane oxidation is predicted to be a capacity of members of Verrucomicrobia, specifically members of the Methylacidiphilales. This reaction involves particulate methane monooxygenase (pMMO-ABC), the genes for which were identified and classified phylogenetically.

One of the more interesting organisms present in the MS4 spring is a Gracilibacteria, which is predicted to have minimal metabolic capacities beyond glycolysis, production of peptidoglycan, and generation of formate, which may be available for use by other community members. Other capacities predicted for this bacterium are the production of riboflavin, amino sugars, RNA degradation, 1C by folate, interconversion of purines and pyrimidines, and biosynthesis of a few amino acids (Fig. 8).

## Discussion

Some springs are hotspots where resources associated with deeply sourced water can sustain chemoautotrophic ecosystems independent of sunlight. We studied two closely spaced but different sites that discharge a mixture of deeply sourced and shallow groundwater, providing microorganisms with reduced compounds and oxygen. Our research integrated geochemical, synchrotron-based spectromicroscopy, metatranscriptomics, and genome-resolved metagenomic data to resolve the network of microorganisms that define the ecosystems. This approach provided insights into organism metabolic capacities and their associations, including those that involve CPR bacteria and the biogeochemical processes that sustain autotrophic ecosystems in the context of their spring-based hydrological setting.

Analysis of the metabolisms of the dominant bacteria in the springs revealed that genes implicated in sulfur cycling are common at both sites (Fig. 8). As expected, the primary energy source is reduced sulfur in the form of sulfide. Overall, the most common sulfur metabolisms are sulfide oxidation, thiosulfate disproportionation, sulfur oxidation, and less commonly sulfite oxidation and sulfate reduction. Sulfide can be oxidized aerobically and in some cases, anaerobically, coupled with nitrate reduction. Our metagenomic analyses suggest that intermediate sulfur compounds and sulfate and sulfide are actively cycled by Campylobacterota (*Sulfurovum*, *Thiovulum*) and Gammaproteobacteria (Thiotrichales and Beggiotales) in the spring communities, probably coupled to nitrogen compound reduction in some microhabitats. Elemental sulfur serves as an energy source stored as sulfur granules as observed by STXM, in *Beggiatoa* [89]. Interestingly, elemental sulfur-bearing granules may serve as an energy source for the growth of *Beggiatoa* and/or *Thiothrix*. The sulfur oxidizers are the primary source of fixed carbon and nitrogen in the ecosystem.

A higher flow rate and a higher concentration of sulfate were observed at MS11 compared to MS4, and the communities have distinct microbial community composition. The MS4 ecosystem is highly diverse and dominated by abundant sulfide-oxidizing Gammaproteobacteria (*Thiothrix*, *Sulfurovum*) and sulfate-reducing Desulfobacterales. The MS11 spring has relatively low diversity and is highly dominated by Campylobacterota (*Sulfurovum*, *Thiovulum*) and Gammaproteobacteria (Thiotrichales and Beggiotales). Our findings are consistent with predictions from studies that indicate that filamentous Campylobacterota dominate biofilms with high sulfide/oxygen (> 150) ratios, whereas Gammaproteobacteria (*Beggiatoa* like) prefer lower (< 75) ratios [9].

We further investigated the metabolic capacities of several CPR bacteria within these communities, as their roles in sulfur-based chemoautotrophic ecosystems remain poorly known. CPR bacteria are often characterized by small genomes and minimal anaerobic fermentative metabolism [90]; however, recent studies have shown auxiliary metabolisms such as the presence of hydrogenases [25, 84], rhodopsin [91], nitrite reductases [92], and F-type ATPase [93], which may contribute to alternative energy conservation and adaptations to different environments and host associations. Notably, we identified genes potentially involved in elemental sulfur reduction (sulfyhydrogenase) and thiosulfate oxidation (persulfide dioxygenase and rhodonase) in the genomes of several CPR bacteria, suggesting a potential new energy generation mechanism for these bacteria. We found that other CPR bacteria from high sulfur environments have the same predicted potential for thiosulfate oxidation, suggesting an important general adaptation of CPR bacteria in sulfur-rich environments.

The most interesting aspect of the current study regards interactions involving CPR bacteria and their host microorganisms. CPR-host associations have rarely been documented, with the exception of oral microbiome-associated Saccharibacteria (TM7) [29, 94], and Actinobacteria further laboratory studies [95] have validated genomic predictions of metabolic interdependency [84]. One study suggested the presence of CPR cells on the surfaces of their Actinobacteria hosts via SEM and showed them to be rod shaped and < 0.2 µm in diameter and ~ 0.5 µm in length [96]. Another study linked *Vampirococcus* with anoxygenic photosynthetic Gammaproteobacteria [97]. Two studies suggest links between Parcubacteria and Archaea, in one case *Methanosaeta* [98] and *Methanothrix* [98]. In the case of the CPR Nealsonbacteria associated with *Methanosaeta*, cryo-TEM imaging indicated that *Methanosaeta*-attached cells are ~ 0.5 µm in diameter. Other cultivation-independent studies have verified that CPR cells are ultra-small and can be better analyzed via filtration through a 0.2-µm pre-filtering [90]. Cryo-TEM imaging and tomographic analyses have documented ultra-small cells directly associated with CPR cells and host bacteria [31, 90]. Generally, these data indicate that CPR cells are a fraction of a micron in length and diameter, consistent with the size for filament-associated ultra-small cells reported here (< 650 nm long, ~ 250 nm wide, the smallest being 290 ± 20 nm long, 120 ± 15 nm wide). In the MS4 biofilms, ultra-small cells were found associated with the surfaces of long filamentous bacteria containing relatively large S^0^ granules as evidenced by STXM. Given that the only abundant filamentous bacteria in these samples are sulfide-oxidizing *Thiothrix*, we predict that some of these tiny episymbiotic cells are CPR bacteria. CPR identifications include Gracilibacteria, Berkelbacteria, Moranbacteria, or Doudnabacteria, based on microbial community abundance information. Unfortunately, our attempts to perform fluorescent in situ hybridization to determine CPR bacterial identity were unsuccessful due to low amount of material, so this inference remains tentative. Future laboratory co-cultivation of *Thiothrix* and their episymbionts may be required to identify the CPR types, so as to better understand the nature of their association (e.g., mutualistic, parasitic). If confirmed, and given the prediction that some CPR bacteria have putative sulfhydrogenases that may produce H_2_S [83, 99], these episymbionts may be involved in cryptic sulfur cycling that also involves sulfur-oxidizing bacteria.

Hydrogen is an important resource in many environments [100], yet little is known about the distribution and importance of hydrogenases in sustaining groundwater microbiomes. The most common chemolithoautotrophs in the Alum Rock spring biofilms are H_2_-oxidizing bacteria, which use H_2_ as an energy source via the enzyme hydrogenase. Specifically, group 3b [NiFe]-hydrogenases are widely distributed in the genomes of many of the microbial community members. These complexes may mediate hydrogen metabolism or the direct hydrogenation of elemental sulfur to hydrogen sulfide [99]. Other hydrogenases of the microbial community members are implicated in hydrogen production and oxidation. Together, these findings suggest that most bacteria in Alum Rock springs cycle hydrogen gas and sulfur compounds, reactions that underpin the biology and geochemistry of this ecosystem.

### Supplementary Information

**Additional file 1:** **Supplementary Fig. 1.** Micro-focused X-ray fluorescence distribution map of sulfur in MS4 filaments at 95 Kelvin. The color intensity scale represents X-ray fluorescence counts.

**Additional file 2:** **Supplementary Fig. 2.** Confocal fluorescence microscopy of MS11 biofilms showing cells treated with SYTOX (blue) for nucleic acid and F-64 (red) for membrane.

**Additional file 3:** **Supplementary Fig. 3.** Sulfur L_2,3_-edges NEXAFS spectra of sulfur granules in MS4 and MS11 biofilms from filaments shown in Fig. 3, compared to an elemental sulfur standard.

**Additional file 4:** **Supplementary Fig. 4.** Scanning transmission x-ray microscopy of filamentous bacteria and small cells in MS11 (A) and MS4 (B-D) biofilms. A) Image at 174 eV (at S L_2_-edge) of an ultra-small cell 477 ± 23 nm long, 218 ± 18 nm wide (blue arrow) in close contact with a cell (black arrow) on the surface of a filament. B) Image at 300 eV (above C K-edge) showing curved-shaped cells near filaments, red arrow points to a cell 764 ± 24 nm long, 309 ± 14 nm wide. C) Cell 656 ± 24 nm long, 284 ± 22 nm wide (red arrow) with extracellular polymeric substances and sulfur granules (white arrow), imaged at 200 eV (above S L_2,3_-edges). D) Episymbiotic cell ~1.1µm long, ~280 nm wide (red arrow), with copious extracellular polymeric substances, imaged at 163.9 eV (at S L_3_-edge), see Fig. 2F-G. An ultra-small cell ~480 nm long, ~270 nm wide (blue arrow) seen in contact with an apparently episymbiotic cell ~2µm long, ~360 nm wide (black arrow). Inset: “Ultra-small” cell 290 ± 20 nm long, 120 ± 15 nm wide (blue arrow), 1µm away from another filament (~3.5 µm wide). The intensity scale corresponds to the optical density. Pixel sizes: 25x25 nm (A-B, D Inset), 40x40 nm (C), 100x100 nm (D).

**Additional file 5:** **Supplementary Fig. 5.** Carbon standard compounds. Bovine serum albumin (BSA, protein), alginate (acidic polysaccharide), agarose (neutral polysaccharide), deoxyribonucleic acid (DNA), 1,2-dipalmitoyl-sn-glycero-3-phosphoethanolamine (PE lipid), sodium bicarbonate (NaHCO_3_). Spectra were normalized at 300 eV. Peaks a: 285.2, b: 288.2, c: 288.6, d: 289.3, e: 285, f: 286, g: 286.7, h: 287.4, i: 289.4, j: 285.1, k: 287.7, l: 288.5, m: 290.2. Vertical dashed line is at 288.2 eV. (see table S2 and references herein).

**Additional file 6:** **Supplementary Fig. 6.** DNA read coverage of all of the genomic bins for each spring.

**Additional file 7:** **Supplementary Fig. 7.** Phylogenetic analyses of RuBisCO-like proteins from the Alum Rock genomic bins. Red circles represent the Alum Rock springs sequences.

**Additional file 8:** **Supplementary Table 1.** Geochemical parameters of the two Alum Rock springs in June 2005. Flow rates given in mL/s, temperature in °C, and concentrations in mg/L.

**Additional file 9:** **Supplementary Table 2.** Major carbon functional groups present in MS4 and MS11 biofilms, the peaks were assigned according to prior work [37, 68, 101–103].

**Additional file 10:** **Table S3.** Nonredundant genomic bins classified as near complete.

**Additional file 11:** **Table S4.** Taxonomic affiliations based on GTDB.

**Additional file 12:** **Table S5.** Genomes coverages (CoverM).

**Additional file 13:** **Table S6.** A. Metabolic expanded.

**Additional file 14:** **Table S7.** Hydrogenases.

## Data Availability

Genomes presented in this manuscript are also made available at https://ggkbase.berkeley.edu/alumrock-genomes.
