# Supplementary material for: Autotrophic biofilms sustained by deeply sourced groundwater host diverse bacteria implicated in sulfur and hydrogen metabolism
Source: Microbiome. 2024 Jan 26;12:15. doi: 10.1186/s40168-023-01704-w (PMC10811913; doi:10.1186/s40168-023-01704-w)
Supplement: Supplementary file 9 — Additional file 8: Supplementary Table 1. Geochemical parameters of the two Alum Rock springs in June 2005. Flow rates given in mL/s, temperature in °C, and concentrations in mg/L. [file 40168_2023_1704_MOESM8_ESM.pdf]

				Cations								Anions					
	**Flow Rate**	**Temp.**	**pH**	Ca^{2+}	Mg^{2+}	Mn^{4+}	Na^{+}	Fe^{3+}	K^{+}	Si^{4+}	Sr^{2+}	Cl^{-}	NO_2^{-}	NO_3^{2-}	PO_4^{2-}	SO_4^{2-}	HS^{-}
**MS4**	169.83	28.80	6.99	83.88	32.21	0.32	643.44	0.06	21.62	10.67	15.6	16.27	2.33	1.93	0.61	106.60	0.29
	[1.76]			[2.37]	[0.93]	[0.016]	[21.28]	-	[0.86]	[0.19]	[.648]	[0.90]	[1.25]	[0.09]	[0.044]	[6.24]	[0.034]
**MS11**	261.75	27.40	7.40	163.20	67.16	0.49	791.89	0.09	24.24	11.95	24.09	29.33	4.33	2.81	0.16	213.30	1.66
	[9.05]			[2.24]	[1.27]	[0.012]	[14.38]	-	[1.34]	[0.17]	[1.26]	[2.62]	[0.47]	[0.50]	[0.06]	[6.24]	[0.29]
