# Supplementary material for: Autotrophic biofilms sustained by deeply sourced groundwater host diverse bacteria implicated in sulfur and hydrogen metabolism
Source: Microbiome. 2024 Jan 26;12:15. doi: 10.1186/s40168-023-01704-w (PMC10811913; doi:10.1186/s40168-023-01704-w)
Supplement: Supplementary file 10 — Additional file 9: Supplementary Table 2. Major carbon functional groups present in MS4 and MS11 biofilms, the peaks were assigned according to prior work [37, 68, 101–103]. [file 40168_2023_1704_MOESM9_ESM.pdf]

	Energy (eV)	Transition	Functional group	Cell	EPS	Filament
a	285.1-285.2	1s→$\pi^*_{C=C}$	unsaturated or aromatic C	y	y	y
b	286	1s→π^*	DNA	y		y
c	286.6-286.7	1s→π^*	ketones, phenols, carbonyl (DNA)	y		y
d	287.4-287.6	1s→π^*<br>1s→σ^*	(DNA), aliphatic C, aromatic carbonyl, aromatic hydroxyl, other oxygenated groups	y	y	y
e	288.2	1s→$\pi^*_{C=O}$	amide carbonyl (peptide bond)	y		y
f	288.7	1s→$\pi^*_{C=O}$	carboxyl (polysaccharide)		y	
g	289.4-289.5	1s→ σ^*<br>1s→π^*	alcohol, aliphatic ether, carbonyl<br>(DNA)	y	y	y
	290.7	1s→$\pi^*_{C=O}$	Carbonates			
	297.4	$2p_{3/2}$ → 3d/σ^*	Potassium L_3			
	299.9	$2p_{1/2}$ → 3d/σ^*	Potassium L_2			
